# The mechanisms of GLP-1 receptor agonists in liver diseases: their multifaceted impact on immune response and metabolic regulation

**DOI:** 10.3389/fphar.2026.1754721

**Published:** 2026-03-19

**Authors:** Zixuan Hu, Dianzhe Tian, Zuyi Yang, Junwei Zhang, Lei Zhang, Yiyao Xu, Xin Lu

**Affiliations:** 1 Department of Liver Surgery, Peking Union Medical College Hospital, Chinese Academy of Medical Sciences and Peking Union Medical College, Beijing, China; 2 Eight-Year Medical Doctor Program, Chinese Academy of Medical Sciences and Peking Union Medical College, Beijing, China; 3 Chinese Academy of Medical Sciences Oxford Institute, University of Oxford, Oxford, United Kingdom

**Keywords:** anti-inflammatory effects, clinical applications, GLP-1RAs, liver injury, metabolic regulation

## Abstract

Glucagon-like peptide-1 receptor agonists (GLP-1RAs) are revolutionizing the management of metabolic and liver diseases, demonstrating effectiveness in controlling blood sugar levels and promoting liver repair. Research suggests that they have protective effects on the liver, demonstrating the potential for fibrosis regression and improved survival rates in patients with advanced liver disease. To synthesize recent advancements in the hepatoprotective effects of GLP-1RAs and their underlying mechanisms, we aimed to provide a comprehensive framework for the development of targeted therapeutics. Our data sources included PubMed and Web of Science, with search terms such as “GLP-1RAs,” “liver,” “MASLD,” “HCC,” “inflammatory,” “microbiota,” “metabolism,” and their combinations. We selected reviews, clinical trials, and basic research articles from the past 5 years. GLP-1RAs provide a comprehensive defense against liver damage by exhibiting anti-inflammatory effects and promoting metabolic changes. They significantly modify the immune microenvironment, lower pro-inflammatory cytokines, and prevent the activation of hepatic stellate cells (HSCs), thereby helping to reduce fibrogenesis. This immune-metabolic modulation enhances their effectiveness in treating chronic liver conditions such as metabolic dysfunction-associated steatotic liver disease (MASLD), fibrosis, and hepatocellular carcinoma (HCC). The combined clinical benefits and mechanistic insights position GLP-1RAs as leaders in addressing glycemic dysregulation and liver diseases, suggesting a transformative approach to the association between metabolic disorders and liver conditions.

## 1 Introduction

**FIGURE 1:**
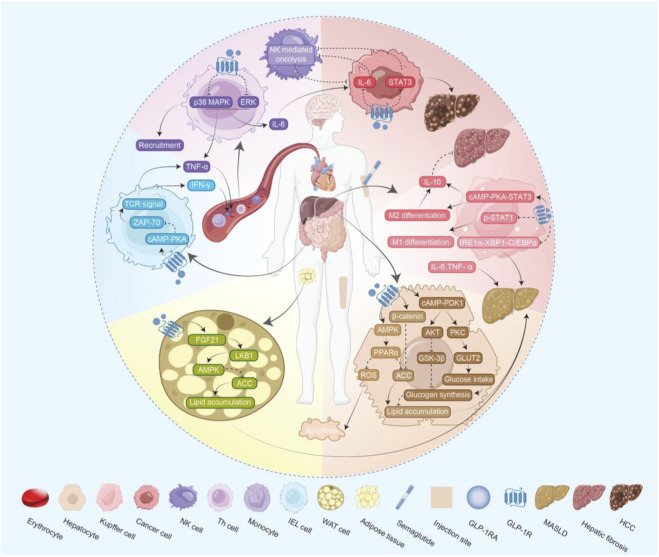
The molecular mechanism map illustrating how GLP-1RAs affect the liver through various pathways. The line with an arrow indicates a stimulation effect, while the dotted line indicates an inhibition effect. GLP-1R: glucagon-like peptide-1 receptor, GLP-1RA: glucagon-like peptide-1 receptor agonist, MASLD: metabolic dysfunction-associated steatotic liver disease, HCC: hepatocellular carcinoma, NK cell: natural killer cell, IEL: intraepithelial lymphocyte, WAT: white adipose tissue, cAMP: cyclic adenosine monophosphate, TCR: T-cell receptor, TNF-α: tumor necrosis factor-α, IFN-γ: interferon-γ PKA: protein kinase A, PKC: protein kinase C, AMPK: adenosine monophosphate-activated protein kinase, PPARα: peroxisome proliferator-activated receptor α, IL-6: interleukin-6, IL-10: interleukin-10, p-STAT1: phosphorylated signal transducer and activator of transcription 1, STAT3: signal transducer and activator of transcription 3, ZAP-70: zeta-chain-associated protein kinase-70, ROS: reactive oxygen species, ACC: Acetyl-CoA carboxylase, FGF21: Fibroblast growth factor 21, LKB1:liver kinase B1, ERK: extracellular regulated protein kinases, IRE1α-XBP1-C/EBPα: Inositol-requiring transmembrane kinase/endonuclease-1α-X-box binding protein 1-CCAAT/enhancer binding protein α, GSK-3β: Glycogen synthase kinase-3 beta, GLUT-2: Glucose Transporter 2.

Liver cirrhosis and HCC are two end-stage situations of liver diseases. The mechanism of their development is closely related to the immunological abnormality (Hammerich and Tacke, 2023) (Figure 1). Nowadays, MASLD, as the most prevalent chronic liver disease, has a rising number of patients, and it is at an early stage of liver disease. Recently, the pharmacotherapy of MASLD mainly focuses on three parts of mechanisms: anti-inflammatory (ASK1 inhibitors), anti-diabetic (GCGR agonists, PPAR agonists), and prevention of lipotoxicity (FXR agonists, FGF21 analogues) (Wei et al., 2024). Added from other targets, the medication for patients would be complex, and the adherence would be low due to the large number of different kinds of medicines. So, whether there is a kind of medicine whose effects can cover as many targets as possible is a good question for liver disease therapy. Fortunately, GLP-1RAs seem to have the potential to become a multi-target medicine.

Glucagon-like peptide-1 (GLP-1) is a polypeptide secreted by intestinal L cells, with its receptors widely distributed throughout the body. These agents regulate glucose metabolism by enhancing insulin secretion, suppressing glucagon release, delaying gastric emptying, promoting weight loss, and improving cardiovascular health (Bendotti et al., 2022). GLP-1RAs have demonstrated potential in treating liver diseases, cirrhosis, respiratory diseases, and neurodegenerative conditions, such as Alzheimer’s and Parkinson’s, in addition to their glucose-lowering effects (Meh et al., 2023; Toki et al., 2021). Ongoing research is ongoing to explore broader therapeutic applications.

Research indicates that GLP-1RAs exert protective effects on the liver through various mechanisms, including anti-inflammatory actions, regulation of hepatic lipid metabolism, and enhancement of hepatocyte survival. These benefits are particularly evident in conditions like nonalcoholic steatohepatitis (NASH), where GLP-1RAs alleviate inflammation and boost insulin sensitivity, leading to improved hepatic glucose management and reduced steatosis (Zheng et al., 2024). Preclinical studies have shown that GLP-1RAs influence essential signaling pathways, including the activation of farnesoid X receptor (FXR) and liver X receptor (LXR), which are crucial in lipid metabolism and inflammation (Errafii et al., 2022). At the cellular level, GLP-1RAs provide liver protection through three main pathways: reprogramming Kupffer cells to reduce inflammation, resetting metabolism via FXR/LXR-mediated lipid efflux, and directly promoting hepatocyte survival through the PI3K/Akt pathway. These processes are particularly effective in treating NASH, with trials demonstrating notable improvements in fibrosis scores. Additionally, GLP-1RAs interrupt the cycle of hepatocyte apoptosis and the activation of hepatic stellate cells, thereby exhibiting anti-fibrotic properties, as evidenced by the decreased collagen deposition in preclinical studies. Beyond NASH, new applications are emerging for HCC, where AMPK activation induced by GLP-1RAs has been shown to have anti-angiogenic effects. Recent findings on extra-pancreatic GLP-1 receptors on liver sinusoidal endothelial cells and hepatic stellate cells help clarify these diverse effects and may explain the survival benefits noted in patients with cirrhosis. As clinical research advances, ongoing trials, such as the European Liver and Intestine Transplant Association (ELITA) and Real Life Intranasal Zolmitriptan Exposure (REALIZE), are set to further explore the role of GLP-1RAs in reversing liver fibrosis, potentially transforming treatment strategies for liver diseases. Their varied effects also indicate their potential in addressing simultaneous metabolic disorders and liver diseases, opening the door for broader indications in liver disease treatment as clinical evidence accumulates (Ibrahim et al., 2024; Yan et al., 2023).

This review examines the impact of GLP-1RAs on liver health and their potential applications in the treatment of liver diseases. By integrating recent studies, we provided an overview of GLP-1RAs in liver pathology and their prospects in clinical practice. Insights from this analysis may lead to novel therapeutic strategies using GLP-1RAs for managing liver diseases associated with metabolic disorders.

## 2 Methods

PubMed was the primary database searched for this review. As the number of articles on GLP-1RAs and liver cancer is low in the 2000–2020 period, but rose significantly after 2020, suggesting a research boom. Eligible publications were limited to articles and reviews written in English and published between 2020 and 2025. Studies not related to the immunological or metabolic interactions between GLP-1 receptor agonists and liver diseases were manually excluded after title and abstract screening. The detailed search terms and selection criteria are described below.

1. The queries for the articles about mechanism of GLP-1RAs in liver disease and the clinical experiments: ((GLP-1RAs[Title/Abstract]) OR (GLP-1R agonists[Title/Abstract]) OR (GLP-1 receptor agonists[Title/Abstract]) OR (GLP-1 receptor[Title/Abstract])) AND ((liver[Title/Abstract]) OR (hepatic[Title/Abstract]) OR (HCC[Title/Abstract]) OR (fibrosis[Title/Abstract]) OR (cirrhosis[Title/Abstract]) OR (NAFLD[Title/Abstract]) OR (MASLD[Title/Abstract])) AND ((inflammation[Title/Abstract]) OR (immun*[Title/Abstract]) OR (T cell[Title/Abstract]) OR (B cell[Title/Abstract]) OR (NK cell[Title/Abstract]) OR (macrophage[Title/Abstract])).
2. The queries for the articles about the gut-liver axis: ((GLP-1RAs[Title/Abstract]) OR (GLP-1R agonists[Title/Abstract]) OR (GLP-1 receptor agonists[Title/Abstract]) OR (GLP-1 receptor[Title/Abstract])) AND ((liver[Title/Abstract]) OR (hepatic[Title/Abstract]) OR (HCC[Title/Abstract])) AND (microbiota[Title/Abstract]).

## 3 The immune and metabolic mechanism of liver injury

### 3.1 The fibrogenic cascade derives from crosstalk between hepatocytes and stromal-immune networks

Hepatocyte and stromal-immune interactions drive liver fibrosis. Injured hepatocytes release Pathogen-Associated Molecular Patterns (PAMPs) and Damage-Associated Molecular Patterns (DAMPs) activate immune responses and recruit Kupffer cells, HSCs, and other immune cells to promote inflammation and fibrosis through cytokine signaling. When hepatocytes are injured or infected, PAMPs and DAMPs trigger immune responses. Upon hepatocyte death, they release P2Y14 ligands that are concentrated in HSCs (Mederacke et al., 2022). Another DAMP, IL-33, activates HSCs either directly or through innate lymphoid cells (ILCs). DAMPs also impact Kupffer cells, activating the nucleotide-binding oligomerization domain-like receptor (NLR) Family, Pyrin Domain-Containing 3 protein (NLRP3), a type of inflammasome leading to IL-1β secretion and inflammation, and pyroptosis, releasing more DAMPs and perpetuating a destructive cycle (Hammerich and Tacke, 2023). HSC activation increases α-smooth muscle actin (αSMA), transforming HSCs into myofibroblasts and contributing to liver fibrosis (Peiseler et al., 2022).

DAMPs and PAMPs activate Kupffer cells via toll-like receptor 4 (TLR4). These cells release the recruiting chemokine CCL-2. Monocytes differentiate into macrophages, which play a crucial role in inflammation and the development of fibrosis. Other immune cells, such as neutrophils, B cells, and T cells, are recruited by chemokines. Blood monocytes differentiate into several types of macrophages: classically activated types (M1) are pro-inflammatory and anti-fibrogenic, whereas alternatively activated M2 types are anti-inflammatory, pro-fibrogenic, and induce M1 cell apoptosis through IL-10 (Kazankov et al., 2019). However, the classification of macrophage polymorphisms needs to be more accurate; for example, a new function-based classification is being applied in some research fields (Mosser and Edwards, 2008). There are some non-M1 and non-M2 “restorative” type macrophages, which have both anti-inflammation and anti-fibrogenic features, highly express matrix metalloproteinase (MMP), and have a LY6C^low^, F4/80^+^ phenotype (Kazankov et al., 2019). Enrichment of that group of macrophages is the primary purpose in resisting liver injury and fibrosis.

Other immune cells play a role in influencing macrophages. B2 cells that participate in the immune system are pro-inflammatory factors that can promote NASH-associated liver fibrosis (Huby and Gautier, 2022). Variable T cells have different effects on liver tissue; T_H_2 and T_H_17 cells have a pro-fibrogenic impact (Hammerich and Tacke, 2023; Huby and Gautier, 2022). IL-17 and IL-22 released by T_H_17 can stimulate the formation of transforming growth factor-β (TGF-β), which promotes fibrosis by activating fibroblasts (Fabre et al., 2018). IL-17 can cause the secretion of pro-inflammatory cytokines, such as tumor necrosis factor-α (TNF-α), and directly promote the activation of HSCs (Meng et al., 2012). Research has revealed an increase in CD8^+^ PD1^+^ T cells in mice and humans with NASH (Pfister et al., 2021). These CD8^+^ T cells attract HSCs with high expression of CCR5 and lead them to apoptosis via the FasL-Fas pathway, which results in the resolution of liver fibrosis (Koda et al., 2021).

### 3.2 The abnormal lipid metabolism and lipid accumulation cause steatosis and lead to inflammation and MASLD

Abnormal lipid metabolism is a crucial cause of MASLD. The overaccumulation of triacylglycerol and cholesterol in the hepatocytes and the overproduction of reactive oxygen species (ROS) both lead to local inflammatory responses. The result of steatosis and inflammation is fibrosis, as we have mentioned in Section 3.1. The extra lipid in the liver comes from several different ways. The intake is the most direct way to get lipids, and another important cause is insulin resistance. Insulin resistance, whether with type 2 diabetes or not, will lead to hyperglycemia, and *de novo* lipogenesis will be stimulated by high blood glucose. It also stopped the transformation of blood glucose into glycogen in muscles. The intermediates from *de novo* lipogenesis in the liver will disrupt mitochondrial function and produce ROS, and then cause endoplasmic reticulum (ER) stress, ultimately leading to inflammation, and the broken cells releasing cytokines will cause more cell death (Huang et al., 2025).

The excess free fatty acids (FFAs) and *de novo* lipogenesis would break the homeostasis of lipid metabolism. Insulin resistance causes stimulation of the lipolysis signal, such as cAMP-PKA, to activate phospho-hormone-sensitive lipase (p-HSL). SREBP1c, LXR, and PPAR-γ, which are great targets for therapy, would be activated in the *de novo* lipogenesis process. The abnormal signal pathways will lead to excessive production of FFAs and ROS in adipocytes and hepatocytes, which is a milestone of the first step to develop MASH (Portincasa et al., 2024). Therefore, stopping the accumulation of FFAs from two directions is of vital importance in the prevention of liver steatosis. By stopping this, the MASLD may be prevented or alleviated if it already exists.

## 4 Mechanisms of hepatoprotection by GLP-1RAs as immune-metabolic modulators

### 4.1 Immunomodulatory mechanism of hepatic injuries by GLP-1RAs

GLP-1RAs reduce inflammation, downregulate pro-inflammatory cytokines, and influence immune cell differentiation via the GLP-1 receptor, which alters macrophage polarization and inhibits fibrosis (Hammerich and Tacke, 2023).

#### 4.1.1 GLP1-RAs attenuate the inflammation in MASLD by downregulating multiple inflammatory factors

In animal models, GLP-1RAs have shown promising results in decreasing the expression of various inflammatory markers, including TLR2 and C-X3-C chemokine receptor 1 (CX3CR1), as well as reducing the expression of genes related to liver fibrosis and fat accumulation. These effects have been observed in NASH, LPS-induced inflammation, and atherosclerosis mouse models (Rakipovski et al., 2018). An experiment in mice also discovered that activation of central GLP-1R leads to downregulation of TNF gene expression, resulting in lower TNF-α expression. This systemic anti-inflammatory effect of TLR agonist-induced inflammation of central GLP-1R is mediated via the α-adrenergic, δ-opioid, and κ-opioid receptor signaling pathways (Wong et al., 2024). GLP-1Rs are expressed in some T-cells. Liraglutide can downregulate the expression of *HMGB-1*, *RAGE*, and *TLR-4* in the livers of mice, causing a reduction in p38 MAPK, p-NF-κB p65, TNF-α, and other inflammatory factors. Liraglutide also induces hepatocyte autophagy by activating the GLP-1R-Akt axis (Elsiad et al., 2025). In peripheral blood mononuclear cells from type 2 diabetes patients, exendin-4 has been confirmed to inhibit the phosphorylation of intracellular ERK and p38 MAPK in CD4^+^ T_H_ cells and monocytes through GLP-1R on these cells (He et al., 2013). These signaling pathways may be related to the other results, such as the reduction of the production of chemokines and pro-inflammatory cytokines, such as IL-6, TGF-β, and TNF-α in TH cells, as well as the expression of adhesin (CD11b) and chemokines, such as CCL-2 (MCP-1) in monocytes (He et al., 2013; Arakawa et al., 2010; Robinson et al., 2015; Hachuła et al., 2024). The cAMP pathway in macrophages of ApoE^−/−^ mice may partially explain this inhibition of chemokine and inflammatory factor secretion. However, the reduction of inflammation is measured by the TNF-α and MCP-1 mRNA levels of isolated cells (Arakawa et al., 2010). A study based on a myocardial infarction mouse model revealed that exendin-4 treatment altered the gene expression of macrophages. The mRNA levels of CD11b and MMP-9 are reduced, and the mRNA levels of IL-10 and IL-1β are increased in macrophages without affecting fibroblasts in the heart (Robinson et al., 2015). Semaglutide can attenuate inflammation in MASLD by downregulating the IRE1α-XBP1-C/EBPα (inositol-requiring transmembrane kinase/endonuclease-1α-X-box binding protein 1-CCAAT/enhancer binding protein α) pathway in macrophages (Hu et al., 2024), the activation of which can increase the production of PGE_2_ (Chopra et al., 2019).

#### 4.1.2 GLP-1RAs regulate the immune cells to protect the liver against fibrosis and neoplasia

Exenatide and liraglutide can elevate cAMP levels and reduce the M1/M2 ratio through the cAMP-PKA-STAT3 pathway, resulting in amelioration of injured liver function and liver inflammation (Li et al., 2022; Li et al., 2019). The cAMP-PKA-STAT3 pathway is related to M2 differentiation, and there is another p-STAT1 protein whose phosphorylation is inhibited by GLP-1RAs, which is an M1 phenotype (Li et al., 2019). Semaglutide and a dual agonist of GLP-1R and glucose-dependent insulinotropic polypeptide receptor, tirzepatide, also positively affect the M1/M2 ratio (Iwamoto et al., 2024). The reduced M1 ratio and normalized Kupffer cell activation are likely to prevent a severe inflammatory response in the liver, thereby decreasing the need for repair. Consequently, there is a lower possibility of initiating fibrosis, and lower expression of some pro-fibrogenic genes was observed in this research (Somm et al., 2021). GLP-1RAs can suppress local inflammation caused by agonistic anti-CD3 antibody through intraepithelial lymphocytes (IELs) (Wong et al., 2022). The activation of cAMP-PKA signaling in gut IELs can inhibit the phosphorylation of Zeta-chain-associated protein kinase-70 (ZAP-70), stopping the signal from TCR, resulting in the inhibition of the immunological response of T cells (Wong et al., 2022; Au-Yeung et al., 2018). The anti-inflammatory and differentiating regulatory effects of GLP-1RAs demonstrate significant potential for alleviating and preventing liver inflammation and fibrosis. However, the absence of experiments employing specific models and measurements of liver fibrosis represents a limitation, as there is scant direct evidence of the anti-fibrotic effects of GLP-1RAs. The effect of decreasing the M1 ratio also suggests that fibrosis may worsen due to the pro-fibrotic M2 cells, so further experiments are needed, especially on the special subtype of M2 cells, to determine whether GLP-1RAs have a positive or negative effect on liver fibrosis and to elucidate the mechanism behind it.

In dealing with HCC, GLP-1RAs also showed promising potential. There is an inflammatory IL-6/STAT3 signaling pathway that is persistently activated in malignancy to protect tumor cells from immune cell attack (Wang et al., 2018). Liraglutide showed anti-tumor effects both *in vitro* and *in vivo*. It suppresses the IL-6/STAT3 pathway, thereby preventing the HCC cells from inhibiting the cytotoxic effect mediated by natural killer cells (NKs) (Lu et al., 2021). In Hepa1-6 inoculated C57BL/6 mice, liraglutide inhibits tumor growth by reducing neutrophil extracellular traps through the inhibition of reactive oxygen species (ROS). The combination of liraglutide and anti-PD-1 can enhance the cytotoxicity of CD8^+^ T cells and induce immunity to tumor rechallenge in Hepa1‐6 allograft mice (Chen et al., 2024). However, a study showed that GLP-1R^pos^ CD3^+^ T cells treated with exendin-4 secreted less IFN-γ, exhibited weaker migration ability, and upregulated genes related to apoptosis, immunoregulation, and immune deficiency, such as CASP3 and CASP7. The effects of apoptosis stimulation were confirmed to occur only in cells expressing GLP-1R in a gain-of-function experiment (Ben et al., 2024). There is more direct animal experimental evidence on the effect of GLP-1RAs on HCC than on fibrosis; however, negative results have also been reported. Therefore, more specific and clinical evidence is needed to confirm our conclusion.

### 4.2 Hepatic metabolic modulation effects by GLP-1RAs

GLP-1RAs replicate the action of the incretin hormone GLP-1, which primarily involves boosting insulin release, reducing glucagon secretion, and increasing feelings of fullness, ultimately leading to improved glycemic control and supporting weight management (Xu et al., 2024). They also play a crucial role in modulating lipid metabolism, reducing hepatic steatosis, and improving liver histology, thereby addressing the underlying pathophysiology of metabolic syndromes (Gu et al., 2023). The adipocyte-to-hepatocyte ratio in the liver significantly influences the hepatic function of gut intraepithelial cells, and lipid accumulation in hepatocytes is partially attributable to insulin resistance and macrophage-mediated adipocyte inflammation (Qi et al., 2024).

Research indicates that GLP-1RAs can ameliorate hepatic steatosis by regulating lipid synthesis pathways and enhancing fatty acid oxidation. Exenatide improved liver fat content and reduced markers of liver injury in animal models fed a high-fructose diet, highlighting the role of β-catenin in mediating these effects (Gao et al., 2020). PPARα mediated GLP-1RAs’ effects on the liver, such as alleviating steatosis and oxidative stress in liver cells, and inhibiting acetyl-CoA carboxylase (ACC) synthesis. The AMPK pathway plays an essential role in the expression of PPARα (Zhou et al., 2020). Liraglutide also reduces HNF1α, PCSK9, and LDLR levels in db/db mice, which strongly inhibits lipid accumulation in the liver and serum (Yang et al., 2018). Liraglutide can increase macrophage fibroblast growth factor 21 (FGF21) secretion and activate FGFR3 in the WAT and macrophages (Zhang et al., 2021). The FGF21-LKB1-AMPK-ACC1 pathway results in the phosphorylation of ACC, inhibiting *de novo* lipogenesis and potentially reducing ferroptosis, a form of cell death. In the T2D mice model, liraglutide showed an effect of alleviating steatosis (Zhang et al., 2021; Guo et al., 2023). Furthermore, GLP-1 and FGF21 fusion proteins have been proven to be more potent than a simple mixture of them in activating downstream pathways. In the HFD mouse model, the fusion protein had a similar effect to GLP-1RAs in lowering serum TC and LDL but exhibited better effects in reducing ALT and AST levels. It also alleviates ballooning and steatosis of hepatocytes (Zhang et al., 2025; Pan et al., 2021). However, a high-fat diet and subsequent development of NASH can downregulate hepatic GLP-1R expression, thereby inducing therapeutic resistance to this medication in certain patients (Svegliati-Baroni et al., 2011; Zhou et al., 2018). Sodium butyrate effectively reversed the downregulation of GLP-1R and demonstrated therapeutic potential for alleviating hepatic steatosis (Zhou et al., 2018).

GLP-1RAs alleviate liver injury by inhibiting hepatocyte apoptosis and promoting the TFEB-dependent transcription of lysosome-related genes, which stimulates hepatocyte autophagy to prevent lipid accumulation (Elsiad et al., 2025; Zhou et al., 2020; Fang et al., 2020). Additionally, clinical trials have shown that GLP-1RAs improve liver histology in patients with MASLD and NASH, as evidenced by reductions in liver fat and inflammation (Gu et al., 2023). The mechanisms involve modulating key transcription factors, such as SREBP-1, which controls *de novo* lipogenesis, along with downregulating enzymes linked to lipid synthesis, such as ACC and FAS (Lyons and Beaudry, 2023). GLP-1RAs are associated with reduced hepatic insulin resistance, which contributes to enhanced glucose homeostasis and improved metabolic profiles in patients with metabolic disorder syndrome (Lin et al., 2024). An *in vitro* experiment demonstrated that GLP-1RA induces the phosphorylation of Akt, PDK-1, and PKC-ζ, which are key proteins involved in the insulin signaling pathway. Insulin-like functions can increase lipolysis and reduce lipid storage in the liver (Gupta et al., 2010).

Exendin-4 can also regulate the transcription of several miRNAs and reduce steatosis in HepG2 cells (Khalifa et al., 2023). miRNA-345-5p, which is upregulated in steatotic cells, targets hypoxia-inducible factor-1 alpha (HIF-1α) and suppresses its expression and subsequent TGFβ/Smad signaling. Blockage of this pathway can curb the activation of HSCs and prevent liver fibrosis (W et al., 2022). The effect of exendin-4 on miRNA-122-5p was downregulated. Research has shown that inhibiting this miRNA attenuates the inflammatory response, oxidative stress, and reverses lipid accumulation in the liver. miRNA-122-5p has been shown to inhibit forkhead box O3 (FOXO3). This protective effect results from the upregulation of FOXO3 (Hu et al., 2022). These findings underscore the therapeutic potential of GLP-1RAs for targeting liver metabolism and mitigating the progression of liver-related metabolic disorders.

## 5 Systemic regulation via gut-liver axis

Intestinal microbiota is a complex and multifunctional system comprising bacteria, viruses, and other microorganisms. Microorganisms in the intestines can pass through the intestinal barrier and enter the portal vein lumen when the permeability of the intestinal barrier decreases. The escaping microorganism ultimately reaches the liver through the bloodstream (Rochoń et al., 2024). Previous studies have demonstrated that chronic endotoxin exposure is related to NASH (Verdam et al., 2011) and is likely due to the activation of TLR4 in immune cells. The low permeability of the intestinal mucosa may be a risk factor for liver disease (Kessoku et al., 2021). Milk-derived extracellular vesicles have been proven to alleviate liver inflammation by restoring gut barrier integrity (Tong et al., 2023). Some immunobiotics can also alleviate NASH and liver fibrosis induced by a methionine-choline-deficient (MCD) diet by reducing intestinal damage and permeability in mice. These immunobiotics have also been shown to reduce plasma T_H_17 levels and inflammatory markers (Kanmani et al., 2024). Reverse research inducing cirrhosis in mice showed that cirrhosis leads to dysfunction of the intestinal barrier, and found that FXR agonists can ameliorate the translocation of gut bacteria to the liver by restoring the integrity of the gut-vascular barrier (Sorribas et al., 2019).

16S rDNA sequencing analysis is a standard method for measuring microbiota diversity, and many interesting conclusions have been drawn from its use. Semaglutide can restore the decreased gut microbiota diversity in *db/db* mice and reverse the changes in specific bacterial levels induced by a high-fat diet (HFD) (Mao et al., 2024; Feng et al., 2024). Semaglutide can reverse the HFD-induced enrichment of bacteria positively correlated with inflammatory factors, such as TNF-α, IL-6, and IL-1β, and can increase those bacteria that are negatively related to inflammatory factors (Feng et al., 2024). The decrease in microbiota diversity in the MCD diet-induced mouse model can also be recovered by liraglutide, along with alleviation of the disrupted gut barrier (Somm et al., 2021). The transmission of the beneficial effect of GLP-1RAs through fecal microbiota transplantation confirmed that GLP-1RAs indeed changed the composition of the gut microbiota in obese mice (Sun et al., 2025). In contrast, specific probiotics can induce the secretion of GLP-1 and indeed ameliorate adipose tissue inflammation (Kim et al., 2025). In addition to the direct impact on the proportion of gut bacteria, GLP-1RAs were found to upregulate tight junction proteins in the intestinal mucosa, which helps prevent the translocation of bacteria to the blood. The adjustment of microbiota leads to the production of N, N-dimethylsphingosine (DMS), which promotes intestinal IL-22-producing ILC3s and alleviates colonic inflammation (Sun et al., 2024). GLP1/2 fusion Fc proteins have been shown to lower weight, alleviate fibrosis, improve tight junctions, and increase microvillus height (Kim et al., 2022). The FXR-activating function of GLP-1RAs also helps protect the gut barrier (Errafii et al., 2022; Sorribas et al., 2019). A lower expression of cecal occluding mRNA after liraglutide treatment was found in a dextran sodium sulfate (DSS)-induced colitis model (Kato et al., 2021), which is contradictory to the results mentioned above (Sun et al., 2024). However, these findings are relatively rare. Overall, the role of the gut-liver axis in treating metabolic dysfunction-associated fatty liver disease by GLP-1RAs is essential.

## 6 Clinical evidence unveils the translational potential of GLP-1RAs

GLP-1RAs notably lower the risk of cirrhosis and HCC in individuals with type 2 diabetes and liver conditions. They also have protective effects on the liver in cases of alcohol-related liver disease, MASLD, and NASH, with a relative risk of 2.48 for resolving NASH. Although fibrosis improvement remains limited, they manage metabolic liver conditions by reducing inflammation and fat deposition, and combining them with anti-inflammatory therapies may enhance the therapeutic efficacy (Yang et al., 2024; Wang et al., 2024; Kanwal et al., 2024; Engström et al., 2024). Table 1 shows representative clinical trials and results of GLP-1RAs in liver diseases.

**TABLE 1:** Clinical studies of GLP-1RA in liver diseases.

Disease	Medication	Control	Dosage	Time	Results	References	NCT
MASLD	Semaglutide	Placebo	0.4 mg/d (after 16 w)	72 w	Sixty‐seven subjects were randomized to once‐daily subcutaneous semaglutide 0.4 mg (n = 34) or placebo (n = 33). Change from baseline in liver stiffness was not significantly different between semaglutide and placebo at week 48 (estimated treatment ratio 0.96 (95% CI 0.89, 1.03; P = 0.2798); significant differences in liver stiffness were not observed at weeks 24 or 72. Reductions in liver steatosis were significantly greater with semaglutide (estimated treatment ratios: 0.70 [0.59, 0.84], P = 0.0002; 0.47 [0.36, 0.60], P < 0.0001; and 0.50 [0.39, 0.66], P < 0.0001) and more subjects achieved a ≥30% reduction in liver fat content with semaglutide at weeks 24, 48 and 72, (all P < 0.001). Decreases in liver enzymes, body weight and HbA1c were also observed with semaglutide	Flint et al. (2021)	03357380
Cirrhosis	Semaglutide	Placebo	2.4 mg/w (after 16 w)	48 w	After 48 weeks, there was no statistically significant difference between the two groups in the proportion of patients with an improvement in liver fibrosis of one stage or more without worsening of NASH (five [11%] of 47 patients in the semaglutide group vs. seven [29%] of 24 in the placebo group; odds ratio 0.28 [95% CI 0.06–1.24; p = 0.087). There was also no significant difference between groups in the proportion of patients who achieved NASH resolution (p = 0.29). Similar proportions of patients in each group reported adverse events (42 [89%] patients in the semaglutide group vs. 19 [79%] in the placebo group) and serious adverse events (six [13%] vs. two [8%])	Loomba et al. (2023)	3987451
Obesity	Semaglutide	Placebo	2.4 mg/w (after 16 w)	44 w	Estimated mean percentage change in bodyweight from baseline to week 44 was −12.1% (SE 0.5) with semaglutide 2.4 mg versus −3.6% (0.7) with placebo (estimated treatment difference −8.5 percentage points [95% CI -10.2 to −6.8]; p < 0.0001). At week 44, the proportion of participants who lost 5% or more of their bodyweight was higher in the semaglutide 2.4 mg group than in the placebo group (203/238 [85%] vs. 36/116 [31%]); odds ratio 13.1 (95% CI 7.4–23.1; p < 0.0001)	Mu et al. (2024)	4251156
NASH	Semaglutide	Placebo	0.1 mg/d	72 w	The percentage of patients in whom NASH resolution was achieved with no worsening of fibrosis was 40% in the 0.1-mg group, 36% in the 0.2-mg group, 59% in the 0.4-mg group, and 17% in the placebo group (P < 0.001 for semaglutide 0.4 mg vs. placebo). An improvement in fibrosis stage occurred in 43% of the patients in the 0.4-mg group and in 33% of the patients in the placebo group (P = 0.48). The mean percent weight loss was 13% in the 0.4-mg group and 1% in the placebo group. The incidence of nausea, constipation, and vomiting was higher in the 0.4-mg group than in the placebo group (nausea, 42% vs. 11%; constipation, 22% vs. 12%; and vomiting, 15% vs. 2%)	Newsome et al. (2021)	02970942
			0.2 mg/d				
			0.4 mg/d				
NASH	Liraglutide	Placebo	1.8 mg/d	48 w	Liraglutide reduced BMI (−1.9 vs. +0.04 kg/m2; p < 0.001), HbA1c (−0.3 vs. +0.3%; p < 0.01), cholesterol-LDL (−0.7 vs. +0.05 mmol/L; p < 0.01), ALT (−54 vs. −4.0 IU/L; p < 0.01) and serum leptin, adiponectin, and CCL-2 (all p < 0.05). Liraglutide increased hepatic insulin sensitivity (−9.36 vs. −2.54% suppression of hepatic endogenous glucose production with low-dose insulin; p < 0.05). Liraglutide increased adipose tissue insulin sensitivity enhancing the ability of insulin to suppress lipolysis both globally (−24.9 vs. +54.8 pmol/L insulin required to ½ maximally suppress serum non-esterified fatty acids; p < 0.05), and specifically within subcutaneous adipose tissue (p < 0.05)	Armstrong et al. (2016)	01237119
T2D	Liraglutide	Placebo	1.8 mg/d	16 w	Liraglutide reduced apoB48 synthesis in chylomicrons by 60% (p < 0.0001) and increased the triglyceride/apoB48 ratio (i.e., the size) of chylomicrons (p < 0.001). Direct clearance of chylomicrons, a quantitatively significant pathway pretreatment, decreased by 90% on liraglutide (p < 0.001). Liraglutide also reduced VLDL1-triglyceride secretion (p = 0.017) in parallel with reduced liver fat	Taskinen et al. (2021)	02765399
MASLD	Exenatide	Glipizide	20 μg/d	6 m	Exenatide can lower the fat contant of liver comparing to glipizide	Kenny et al. (2010)	01951651
MASLD	Exenatide+pioglitazoe	Pioglitazone	20 μg/d	12 m	In type 2 diabetes mellitus, combined pioglitazone and exenatide therapy is associated with a reduction in plasma FGF21 levels, as well as a greater decrease in hepatic fat than that achieved with pioglitazone therapy. In DIO mice, exendin-4 treatment reduces hepatic triacylglycerol and FGF21 protein, and enhances hepatic AMPK phosphorylation, suggesting an improvement of hepatic FGF21 resistance	Samson et al. (2011)	01432405

### 6.1 MASLD and NASH

Studies have shown that GLP-1RAs reduce hepatic fat content and improve liver histology in patients with MASLD, decreasing steatosis, ballooning necrosis, and liver enzyme levels. Their mechanisms enhance insulin sensitivity, reduce hepatic fat synthesis, improve lipid metabolism, and alleviate steatosis (Yabut and Drucker, 2023). By reducing inflammation and fibrosis, standardized diagnosis and assessment of MASLD encompass the quantitative evaluation of hepatic steatosis and fibrosis, as well as cardiovascular risk assessment. Treatment should primarily focus on weight reduction and the improvement of insulin resistance to prevent and treat metabolic syndrome, type 2 diabetes, and its complications. GLP-1RAs have been shown to improve diabetes, promote weight loss, and reduce hepatic fat content (Yao et al., 2024). Metabolic surgery and liver transplantation are therapeutic options for severe cases. Research indicates that although semaglutide did not significantly improve hepatic fibrosis, it had positive effects on hepatic steatosis, weight control, and diabetes-related parameters, offering a new therapeutic direction for patients with NASH and compensated cirrhosis (Flint et al., 2021; Loomba et al., 2023). Therefore, the management of MASLD requires a multidimensional and comprehensive treatment strategy, particularly integrated management of metabolic syndrome and type 2 diabetes.

A study showed that the long-term use of GLP-1RAs could reduce the risk of major adverse liver events (MALE) in patients with type 2 diabetes. After 10 years of treatment, the incidence of MALE was significantly lower in the GLP-1RAs group than in the non-GLP-1RAs group (Celsa et al., 2025). However, intention-to-treat analysis showed no significant difference between the two groups, indicating the need for further large-scale randomized controlled trials to confirm these findings. GLP-1R/GCGR (glucagon receptor) dual agonists have marked significant advancements in the treatment of weight loss, glycemic control, and NASH. By simultaneously activating GLP-1R to boost insulin secretion and GCGR to stimulate fat oxidation, these medications outperform single-target therapies in enhancing metabolism (Boland et al., 2020). When used for NASH, dual agonists decrease hepatic fat levels, enhance insulin sensitivity, and combat hepatic pathological complications (Monfeuga et al., 2024). Clinical trials have shown that dual agonists significantly reduce hepatic fat and NASH progression. The Mazdutide trial also demonstrated that dual agonists could reduce hepatic fat in obese Chinese patients (Ji et al., 2023). As research progresses, these dual agonists offer better therapeutic hope for patients with metabolic disorders and NASH (Sanyal et al., 2024).

### 6.2 Liver fibrosis/Cirrhosis

The progression of fibrosis generally stems from the activation of pro-fibrogenic immune cells, such as M2 cells, which follow inflammatory immune cells that cause injury. Theoretically, GLP-1RAs can worsen liver fibrosis by stimulating M2 cell differentiation. Unlike the steatosis, clinical results about existing fibrosis are not satisfactory. Some studies have only indicated that GLP-1RAs may significantly reduce liver fibrosis markers and improve liver function in patients with chronic liver disease. A cohort study found that GLP-1RAs were associated with a reduced risk of hepatic decompensation and mortality in individuals with type 2 diabetes cirrhosis (Yen et al., 2024). The anti-fibrotic effects of GLP-1RAs may arise from their ability to improve metabolic control, reduce body weight, and directly affect HSCs. This suggests that a decrease in the proportion of M1 cells does not necessarily correspond to an increase in the proportion of classical M2 cells. Rather, there appears to be an increase in the number of non-classical anti-inflammatory and anti-fibrogenic cells. As a result, single-cell sequencing of liver macrophages can provide clearer insights into macrophage distribution within the liver effectively (Li et al., 2022; Li et al., 2019). But there are also many clinical studies that gave a neutral result in improving cirrhosis, and the side effects of this type of medication did occur (Table 1). Positive results were often shown in the patients who did not have cirrhosis, which lowers the risk instead of reversing the fibrosis. This may remind us that the anti- and pro-fibrosis effects of GLP-1RAs may coexist and manifest through different mechanisms. In addition, combining GLP-1RAs with other therapeutic agents, such as sodium-glucose cotransporter 2 inhibitors (SGLT2i), has shown synergistic effects in reducing liver stiffness and improving overall liver health (Lin et al., 2024).

### 6.3 HCC

Preliminary evidence suggests that GLP-1RAs may provide protective effects against HCC development. GLP-1RAs can reduce the IL-6/STAT3 signaling pathway and enhance the cytotoxicity of NK and CD8^+^ T cells, potentially promoting the elimination of cancer cells (Lu et al., 2021; Chen et al., 2024). GLP-1RA also causes apoptosis of CD3^+^ T cells (Ben et al., 2024), contrary to NK stimulation. Clinical experiments have demonstrated the dual positive effect of GLP-1RAs on HCC and cirrhosis, suggesting its logical immune system effects (Yang et al., 2024; Wang et al., 2024; Kanwal et al., 2024; Engström et al., 2024). The details of these effects and their relationships require further exploration. For example, the CD3^+^ T cell experiment, for which a few similar studies have shown the same result, can be further explored by using additional markers to distinguish the smaller groups of T cells and observe their apoptosis ratio (Ben et al., 2024). A systematic review indicated that patients treated with GLP-1RAs had a significantly lower risk of incident HCC compared to those receiving other antidiabetic medications, such as insulin and sulfonylureas (Sha et al., 2024). The mechanisms proposed for this protective effect include modulation of inflammatory pathways, reduction of oxidative stress, and improvement of metabolic parameters that contribute to liver carcinogenesis (Arvanitakis et al., 2022). Furthermore, GLP-1RAs have been shown to positively influence body weight and metabolic control, which are critical factors in managing patients at risk of developing HCC (Wang et al., 2024). Further research is needed to clarify the long-term effects of GLP-1RAs on liver cancer outcomes and their role in cancer prevention in high-risk populations. The potential of GLP-1RAs to reduce the risk of liver cancer is an exciting frontier in liver disease management.

## 7 Comparison with other drugs

Some clinical studies have used other metabolic medications and compared their effects on liver diseases to those of GLP-1RAs. We found that the GLP-1RAs showed identified advantages in liver protection, especially in the replacement of insulin or sulfonylurea. GLP-1-based multi-target agonists demonstrate more comprehensive therapeutic efficacy compared to a single drug, pointing out the future direction of medication application. These findings provide crucial evidence for developing individualized treatment plans tailored to the clinical risks associated with different liver diseases in patients with type 2 diabetes.

### 7.1 Comparing with the conventional hypoglycemic agents

Compared with DDP-4i, GLP-1RAs showed better results in reducing the risk of cirrhosis and mortality (Kanwal et al., 2024). Compared with long-acting insulin, GLP-1RA therapy reduces the risk of composite liver disease (cirrhosis or hepatocellular carcinoma) in patients with type 2 diabetes by 44%, with a 41% reduction in cirrhosis risk and a 53% reduction in hepatocellular carcinoma risk. This supports prioritizing GLP-1RA over insulin in patients requiring intensive glycemic control, potentially offering additional hepatoprotective benefits (Yang et al., 2024). A meta-analysis showed that sulfonylurea use is associated with an increased risk of HCC, while GLP-1RAs can lower the risk of HCC (Arvind et al., 2021). It is worth noting that research comparing GLP-1RAs with six anti-diabetes drugs revealed that there is no significant difference in lowering the risk of HCC between GLP-1RAs and metformin or DDP-4i (Wang et al., 2024).

### 7.2 Comparing with the novel metabolic target drugs

As the FXR-activating function of GLP-1RAs, FXR agonists may have the same effect as GLP-1RAs (Errafii et al., 2022). The combination of GLP-1RAs and FXR agonist therapy is under investigation. A meta-analysis of multiple drugs compared GLP-1RAs and other 4 metabolic medications (THR-β agonists, FGF-21 analogues, GLP-1-based polyagonists, and Pan-PPAR agonists). The conclusion is that GLP-1-based polyagonists show the best overall efficacy, including MASH resolution, improvement in fibrosis, and reduction in the liver fat fraction (Lin et al., 2024). The multiple-target drug had better effects, while the single-target drugs showed a significant improvement in steatosis and glucose metabolism, particularly with GLP-1RAs.

In reducing the risk of major adverse liver outcomes and HCC, large-scale observational studies and meta-analyses have demonstrated that GLP-1RA and SGLT2i exhibit comparable protective effects, with both showing significant superiority over traditional hypoglycemic agents such as DPP-4i and sulfonylureas. Studies have directly compared the effects of the two treatments on the risk of MASLD/MASH, with results showing that both can improve hepatic steatosis and liver enzymes, though the differences may not be statistically significant. However, combination therapy demonstrates greater potential. A *post hoc* analysis in Japan indicated that the combination of SGLT2i and GLP-1RA was superior to monotherapy in improving liver function parameters (ALT, AST), hepatic steatosis index (HSI), and fibrosis index (FIB-4), suggesting complementary mechanisms between the two. Combined use may represent a more optimal strategy (Tsuriya et al., 2025; Chang et al., 2024).

## 8 Limitations

This review has several limitations that warrant consideration. First, a substantial proportion of the mechanistic evidence is derived from animal models and *in vitro* studies, which may not fully capture the complexity of human liver physiology and disease pathogenesis. Second, most clinical trials assessing GLP-1 receptor agonists in liver diseases have relatively short follow-up periods, resulting in limited evidence regarding long-term hepatic outcomes. Third, although existing studies consistently demonstrate beneficial effects on hepatic steatosis and inflammation, the lack of head-to-head mechanistic and biomarker-based comparisons restricts the ability to distinguish between different GLP-1RAs or combination therapies in terms of their molecular pathways, immune–metabolic regulatory efficiency, and predictive biomarkers of therapeutic response. Collectively, these limitations underscore the need for longer-term clinical studies, human-based mechanistic investigations, and well-designed comparative analyses to more fully validate the therapeutic potential and optimize the clinical application of GLP-1RAs in liver diseases.

## 9 Conclusion

GLP-1RAs, a new type of diabetes medication, have shown great potential in treating liver diseases. It can affect the liver microenvironment through GLP-1R in the immune system, adipose tissue, intestines, and the liver. It can regulate the immune system to reduce inflammation, and some clinical results declare it has great potential to prevent fibrosis and HCC. Most of the pathways in this part involve cytokines, starting from MAPK, TCR, or cAMP-PKA signaling, which results in an increase in anti-inflammatory cytokines and a decrease in pro-inflammatory cytokines. In adipose tissue, GLP-1RAs affect the ACC. It mainly inhibits liver lipid accumulation, which benefits patients with type 2 diabetes. Local effects in the liver are divided into two parts. One is through AMPK or β-catenin, which reduces lipid accumulation and prevents hepatocyte apoptosis. The other is an insulin-like effect that starts with the cAMP-PDK1 signal, which stimulates the absorption of blood glucose. However, owing to the inhibition of ACC, this effect does not promote liver steatosis and can reduce blood glucose levels.

GLP-1RAs affect different types of liver disease. The insulin-like effect and inhibition of ACC benefit most patients with MASLD. It can cause noticeable weight loss and control blood glucose levels, which are helpful in T2D patients. Recent research has shown that the GLP-1R/GCGR dual agonists can stimulate the oxidation of lipids; the combined therapy shows better effects on blood glucose control and weight loss (Boland et al., 2020). The long-term use of GLP-1RAs can reduce the risk of MALE in TD patients, thereby improving prognosis. Theoretically, GLP-1RAs can worsen liver fibrosis by modulating the immune system, which lowers the M1 ratio and elevates M2 cells. However, in some clinical studies, these results positively affected liver fibrosis. Although the impact of cirrhosis needs further investigation, the M2 cells require additional studies to separate them into different groups. We hope that anti-inflammatory and anti-fibrotic cells will be identified. Combined therapy for fibrosis also achieved excellent results. In HCC treatment, GLP-1RAs play a role in the immune system, and the activation of NK cells can release the existing HCC; however, the preventive role of this type of drug seems more critical. Several clinical studies have revealed the role of GLP-1RAs in lowering the risk of HCC in patients with TD. However, the details of the molecular mechanism remain unclear, and more research is needed to determine the modulation or interaction of GLP-1RAs with the immune system.
